# Accumulation of metals in GOLD4 COPD lungs is associated with decreased CFTR levels

**DOI:** 10.1186/1465-9921-15-69

**Published:** 2014-06-23

**Authors:** Fatemat Hassan, Xiaohua Xu, Gerard Nuovo, David W Killilea, Jean Tyrrell, Chong Da Tan, Robert Tarran, Philip Diaz, Junbae Jee, Daren Knoell, Prosper N Boyaka, Estelle Cormet-Boyaka

**Affiliations:** 1 Department of Veterinary Biosciences, The Ohio State University, 1925 Coffey Road, Columbus, OH 43210, USA; 2 Phylogeny Inc., Columbus, OH, USA; 3 Nutrition and Metabolism Center Children's Hospital Oakland Research Institute, Oakland, CA, USA; 4 Cystic Fibrosis/Pulmonary Research and Treatment Center, University of North Carolina, Chapel Hill, NC, USA; 5 Department of Internal Medicine, Division of Pulmonary, Allergy, Critical Care and Sleep Medicine, The Ohio State University, Columbus, OH, USA; 6 Current address: Pediatric Department, Brookdale University Hospital and Medical Center, Brooklyn, NY 11212, USA

**Keywords:** COPD, CFTR, Cigarette smoke, Cadmium, Manganese, Lung epithelial cells

## Abstract

**Background:**

The Cystic Fibrosis Transmembrane conductance Regulator (CFTR) is a chloride channel that primarily resides in airway epithelial cells. Decreased CFTR expression and/or function lead to impaired airway surface liquid (ASL) volume homeostasis, resulting in accumulation of mucus, reduced clearance of bacteria, and chronic infection and inflammation.

**Methods:**

Expression of CFTR and the cigarette smoke metal content were assessed in lung samples of controls and COPD patients with established GOLD stage 4. CFTR protein and mRNA were quantified by immunohistochemistry and quantitative RT-PCR, respectively. Metals present in lung samples were quantified by ICP-AES. The effect of cigarette smoke on down-regulation of CFTR expression and function was assessed using primary human airway epithelial cells. The role of leading metal(s) found in lung samples of GOLD 4 COPD patients involved in the alteration of CFTR was confirmed by exposing human bronchial epithelial cells 16HBE14o- to metal-depleted cigarette smoke extracts.

**Results:**

We found that CFTR expression is reduced in the lungs of GOLD 4 COPD patients, especially in bronchial epithelial cells. Assessment of metals present in lung samples revealed that cadmium and manganese were significantly higher in GOLD 4 COPD patients when compared to control smokers (GOLD 0). Primary human airway epithelial cells exposed to cigarette smoke resulted in decreased expression of CFTR protein and reduced airway surface liquid height. 16HBE14o-cells exposed to cigarette smoke also exhibited reduced levels of CFTR protein and mRNA. Removal and/or addition of metals to cigarette smoke extracts before exposure established their role in decrease of CFTR in airway epithelial cells.

**Conclusions:**

CFTR expression is reduced in the lungs of patients with severe COPD. This effect is associated with the accumulation of cadmium and manganese suggesting a role for these metals in the pathogenesis of COPD.

## Introduction

Chronic obstructive pulmonary disease (COPD) is the third leading cause of death in the US since 2008 [1]. The two major clinical phenotypes in COPD in the lung are emphysema and chronic bronchitis. Chronic bronchitis is a disease of the airways while emphysema characterizes the airspaces that are involved in gas exchange [2,3]. The severity of obstruction or airflow limitation in COPD is classified based on the Global Initiative for Chronic Obstructive Lung Disease (GOLD) criteria with GOLD 1 being mild COPD and GOLD 4 very severe COPD.

CFTR is a chloride channel that primarily resides in the apical membrane of epithelial cells. CFTR plays a major role in maintaining ASL volume and hence maintaining normal physiology of the lung. Mutations of the CFTR chloride channel result in cystic fibrosis, which is an autosomal recessive disorder common in Caucasians and characterized by thick viscid mucus secretion blocking the airways leading to recurrent infections by resistant organisms [4]. In the past few years, increasing evidence has linked cigarette smoke exposure and dysregulation of ion transport to reduced expression of the CFTR protein and mucus dehydration [5-8]. Thus, it has been hypothesized that cigarette smoke-induced CFTR dysfunction contributes to the development of chronic bronchitis.

We and others recently reported that heavy metals such as arsenic and cadmium down-regulate the expression of the CFTR protein in human airway epithelial cells [9,10]. Since cigarettes contain high amounts of metals including cadmium and arsenic (0.87 and 0.17 μg/g) [11], we investigated their contribution in cigarette smoke-induced down-regulation of CFTR. Due to the central role played by CFTR in the lung, the present study investigated whether the expression of CFTR was affected or not in the lung of COPD patients with a history of smoking. Herein we show that CFTR protein is decreased in bronchial epithelial cells from patients with severe COPD (GOLD 4). We also identified heavy metals present in cigarette smoke as major down-regulators of CFTR expression.

## Materials and methods

### Isolation and culture of human bronchial epithelial cells

Primary human bronchial epithelial cells (HBEC) were isolated from excess donor tissue obtained at the time of lung transplantation under a protocol approved by UNC Medical School IRB. Primary HBEC were cultured as previously described and studied when fully differentiated [8,12]. Human bronchial epithelial cells 16HBE14o- that express endogenous CFTR, kindly provided by Dr. Gruenert, were cultured in Minimum Essential medium (MEM) supplemented with 10% fetal bovine serum, 1% L-glutamine, and 1% penicillin/streptomycin in a humidified CO_2_ incubator (37°C, 5% CO_2_). The flasks and plates were coated with an extracellular matrix cocktail comprised of bovine serum albumin (Invitrogen), human fibronectin (BD Laboratories), and collagen (BD Laboratories).

### Subjects and sample collection

Human lung samples were obtained from the Lung Tissue Research Consortium (LTRC, NIH) approved project (Concept Sheet #09-99-0017). The LTRC Patients were classified into two groups based on lung function tests with GOLD 4 having an FEV_1_/FVC < 70%, FEV_1_ < 30% predicted or <50% normal with chronic respiratory failure, and GOLD 0 being asymptomatic with normal lung function (Table 1). Patients from both groups had a history of smoking except one patient in control group (GOLD 0).

**Table 1:** Anthropometric characteristics and pulmonary function data of human subjects

**Variables**	**GOLD 0**	**GOLD 4**	***p *** **value**
	**(n = 9)**	**(n = 11)**	
Age, years	65.1 ± 7.4	55.5 ± 1.9	0.09 (n.s.)
Male/Female gender	5/3	4/6	-
Smoking, pack-year	22.5 ± 12.1	58 ± 10.8	0.02*
Stop years	32.8 ± 8.9	7.2 ± 7.7	3.4 × 10^−6^**
FEV1, % predicted	103.4 ± 7.3	18.1 ± 1.2	3.47 × 10^−10^**
FVC, % predicted	100.8 ± 8.1	48.5 ± 3.7	6 × 10^−10^**
FEV1/FVC	75 ± 1.9	32 ± 3.9	4 × 10^−8^**

Data are presented as mean ± SEM; n.s., non significant; *p < 0.05, **p < 0.01.

### Whole cigarette smoke and cigarette smoke extract (CSE) preparation

HBECs were exposed to whole cigarette smoke (CS) with a LM1 smoke engine (Borgwaldt) calibrated to deliver a volume/surface area of CS that approximates *in vivo* exposure [8]. HBECs were serosally perfused with KBR solution during the whole cigarette smoke exposure period. For chronic smoke exposure (5 days) HBECs were exposed to smoke from 2 cigarettes and replaced in the incubator in fresh media between smoke exposures. Smoke was generated according to ISO standards (1 puff = 2 second/35 ml draw). Two cigarettes approximately equaled 30 puffs of smoke.

Cigarette smoke from one non-filtered cigarette was bubbled using a peristaltic pump apparatus into 10 ml of complete culture media (Minimum Essential Medium with 10% fetal bovine serum, 1% L-glutamine, and 1% penicillin/streptomycin), which was designated as 100% CSE. The CSE was prepared from commercial Camel cigarettes (RJ Reynolds). Each experiment has been performed with at least three separate preparations of CSE. Non-filtered cigarettes were chosen since filters remove the particulate fraction which contains metals [13].

### Immunohistochemistry

Immunostaining of CFTR in formalin fixed, paraffin embedded 4 μm thick sections was performed using the Ventana Benchmark LT System and the universal fast red and DAB (red and brown color, respectively) detection systems. The polyclonal rabbit CFTR antibody (Abcam, Cambridge, MA) was used at a dilution of 1:125 for human lung tissues. Optimal conditions included antigen retrieval for 30 min at 95°C using the Ventana Cell Conditioning Antigen retrieval solution #1. This solution is the standard pH 8.5 Tris-EDTA antigen retrieval solution. Negative control was performed by adding rabbit non-immune IgG. Lung sections that did not have bronchial epithelium were excluded. Each slide (representing one patient) was given a score from 1–3 by three independent pathologists/trained researchers (blinded to the results) based upon quantification of the CFTR staining in terms of intensity, localization and number of positive cells.

### ASL height measurements

The height of the ASL was measured as previously described [14]. Briefly, PBS containing 2 mg/ml rhodamine-dextran (10 kDa; Invitrogen, USA) was added to the apical side of polarized human bronchial epithelial cells. A total of five predetermined points (one central, four 2 mm from the edge of the culture) were XZ scanned using a confocal microscope (Leica SP5; glycerol 63× immersion lens). Between time points, the cultures were returned to a humidified CO_2_ incubator and incubated at 37°C in presence of 5% CO_2_. In order to prevent evaporation of the ASL, perfluorocarbon was added apically during imaging.

### Surface biotinylation

Apical membrane proteins were biotinylated as previously described [15]. Briefly, polarized human bronchial epithelial cells were washed three times with PBS supplemented with 1 mM MgCl_2_ and 1 mM CaCl_2_ (PBS-CM). Sulfo-NHS-biotin (0.5 mg/ml) in borate buffer (85 mM NaCl, 4 mM KCl, 15 mM Na_2_B_4_O_7_, pH 9) was applied onto the apical membrane and incubated for 30 min with gentle agitation. PBS-CM supplemented with 10% (v/v) FBS was added to the basolateral bath to prevent biotinylation of basolateral proteins. Cells were lysed in lysis buffer (0.4% sodium deoxycholate, 1% NP-40, 50 mM EGTA, 10 mM Tris-Cl, pH 7.4 and Protease inhibitor) and protein concentration was determined by BCA assay. Three hundred micrograms of total protein were incubated overnight with 100 μl of neutravidin agarose beads at 4°C with agitation. Biotinylated proteins bound to beads were washed three times with lysis buffer and eluted in 30 μl of Laemmli buffer supplemented with 10% (v/v) β-mercaptoethanol by first incubating at room temperature for 10 minutes, followed by heating at 95°C for another 10 minutes.

### Western blotting

Cells were lysed in phosphate buffered saline (PBS) containing 0.2% Triton-X100 and a cocktail of protease inhibitors (Roche). Proteins were detected as previously described using the specific primary antibody diluted at 1:2,000 for C-CFTR (R and D Systems), 1:1000 for Na^+^/K^+^-ATPase (Santa Cruz Biotechnology) or 1:10,000 for β-actin (Santa Cruz Biotechnology) [9,16].

### LDH cytotoxicity assay

Lactate dehydrogenase (LDH) released into the medium was measured using the Tox7 kit (Sigma-Aldrich) by following the manufacturer’s instructions. Results are expressed as percent of total LDH content which was obtained using 1% Triton X-100.

### Quantitative RT-PCR (qRT-PCR) analysis

Quantitative RT-PCR was employed to measure the transcript levels of the *cftr* gene and was performed as previously described [9,17]. RT-PCR for amplifying transcripts of the *cftr* gene was performed at least three times to confirm the accuracy of the results. The CFTR mRNA was normalized to the expression of the housekeeping gene (CAP-1) and expressed as relative copy number (RCN). RCN = 2^ΔCt^ × 100 where ΔCt = Cycle threshold (Ct) of CFTR - Ct of the house keeping gene (CAP-1).

### Elemental analysis

The metal content of tissue samples was determined by inductively-coupled plasma atomic emission spectrometry (ICP-AES). Flash frozen tissues obtained from the LTRC were transferred to pre-weighed polypropylene tubes and desiccated for 12–16 hours at 60°C. The dried pellets were weighed and dissolved in OmniTrace 70% HNO_3_ (EMD Chemicals) overnight at 60°C with slow orbital shaking. Tissue acid lysates were then diluted to 5% HNO_3_ with OmniTrace water (EMD Chemicals), clarified by centrifugation (3000 × g for 10 min), and introduced via a pneumatic concentric nebulizer using argon carrier gas into a Vista Pro ICP-AES (Varian Inc) within 1–2 hours of sample preparation as previously described [18]. All reagents and plasticware were certified or routinely tested for trace metal work. Elemental content data was summarized using native software (ICP Expert; Varian, Inc) and normalized to dry weight of tissue sample.

### Statistical analysis

Data are expressed as mean ± standard error of the mean (SEM) or median with 25% and 75% quartiles of at least 3 independent experiments. Statistically significant differences were assessed using Student’s *t*-test and Mann–Whitney *U* test for analyzing immunohistochemistry results. p values < 0.05 were considered significant.

## Results

### Cigarette smoke alters ASL in primary human bronchial epithelial cells

**Figure 1:**
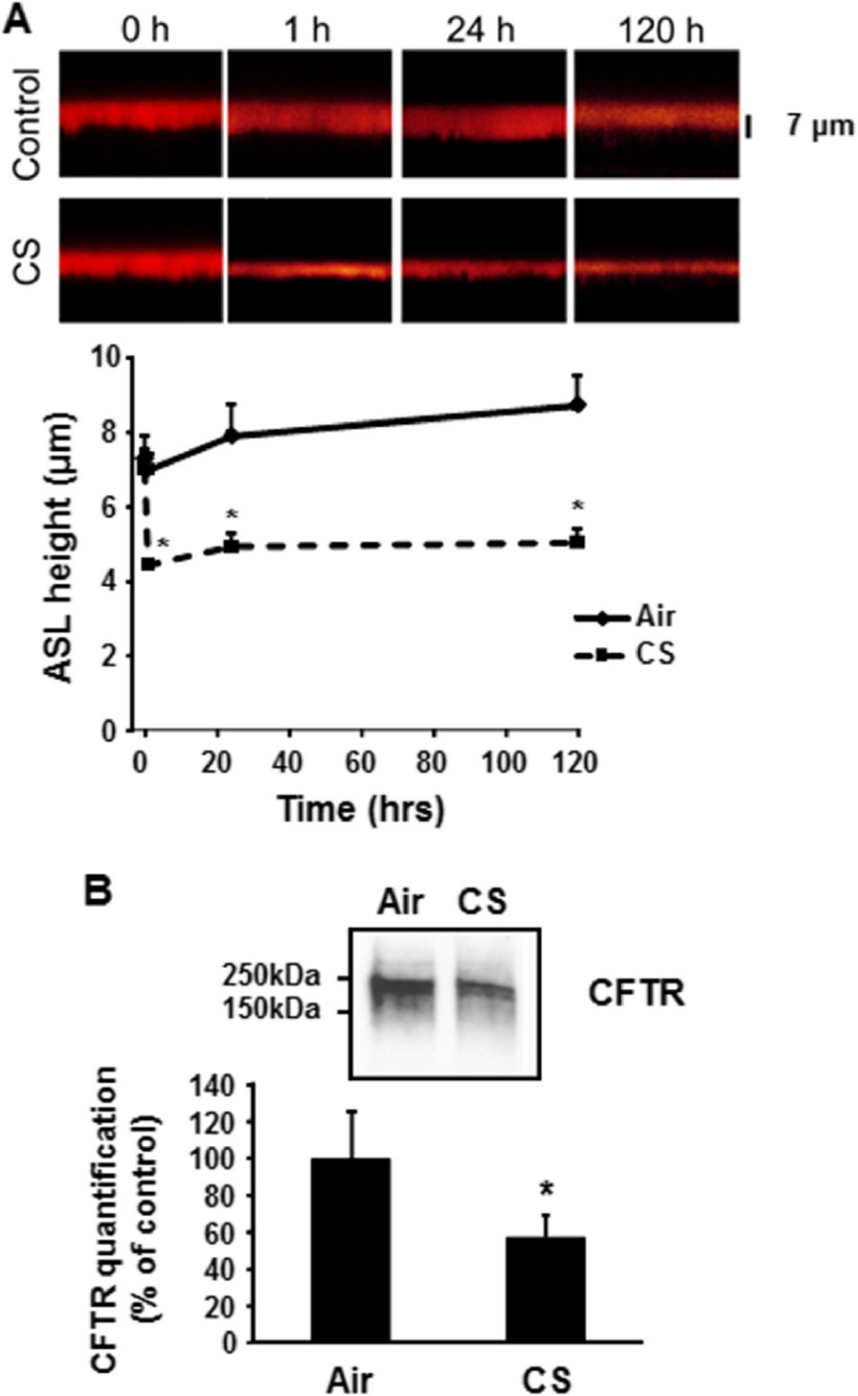
**Chronic exposure to cigarette smoke (CS) decreases airway surface liquid (ASL) height.** Primary human airway epithelial cells from 4 donors (n = 8) were exposed to 30 puffs of whole cigarette smoke (2 cigarettes) every day for 5 days (120 hrs). **(A)** ASL height was measured one hour after each exposure to CS. ASL height was undisturbed over the course of the reading. *p < 0.05. **(B)** CFTR present at the plasma membrane was detected by immunoblotting after biotinylation of cell surface proteins (see Methods).

CFTR is a chloride channel which regulates hydration of the airway surface liquid (ASL) layer [19]. Absence of functional CFTR from the plasma membrane of bronchial epithelial cells results in impaired mucociliary clearance due to reduced airway surface liquid. Previous report showed that acute exposure of primary bronchial epithelial cells to cigarette smoke exerts a transient decrease in ASL height [8]. In order to mimic chronic smoking, human primary bronchial epithelial cells were grown in air/liquid interface and subjected to cigarette smoke for up to 120 hours. The height of the ASL was monitored and decreased significantly upon exposure to cigarette smoke (Figure 1A). To exert its role as chloride channel, the CFTR protein has to be present at the plasma membrane of airway epithelial cells. Exposure to cigarette smoke lead to significant loss of plasma membrane CFTR (Figure 1B). Taken together, our results show that cigarette smoke decreases the expression of CFTR resulting in reduced ASL.

### Cigarette smoke decreases the expression of the CFTR protein in human bronchial epithelial cells

**Figure 2:**
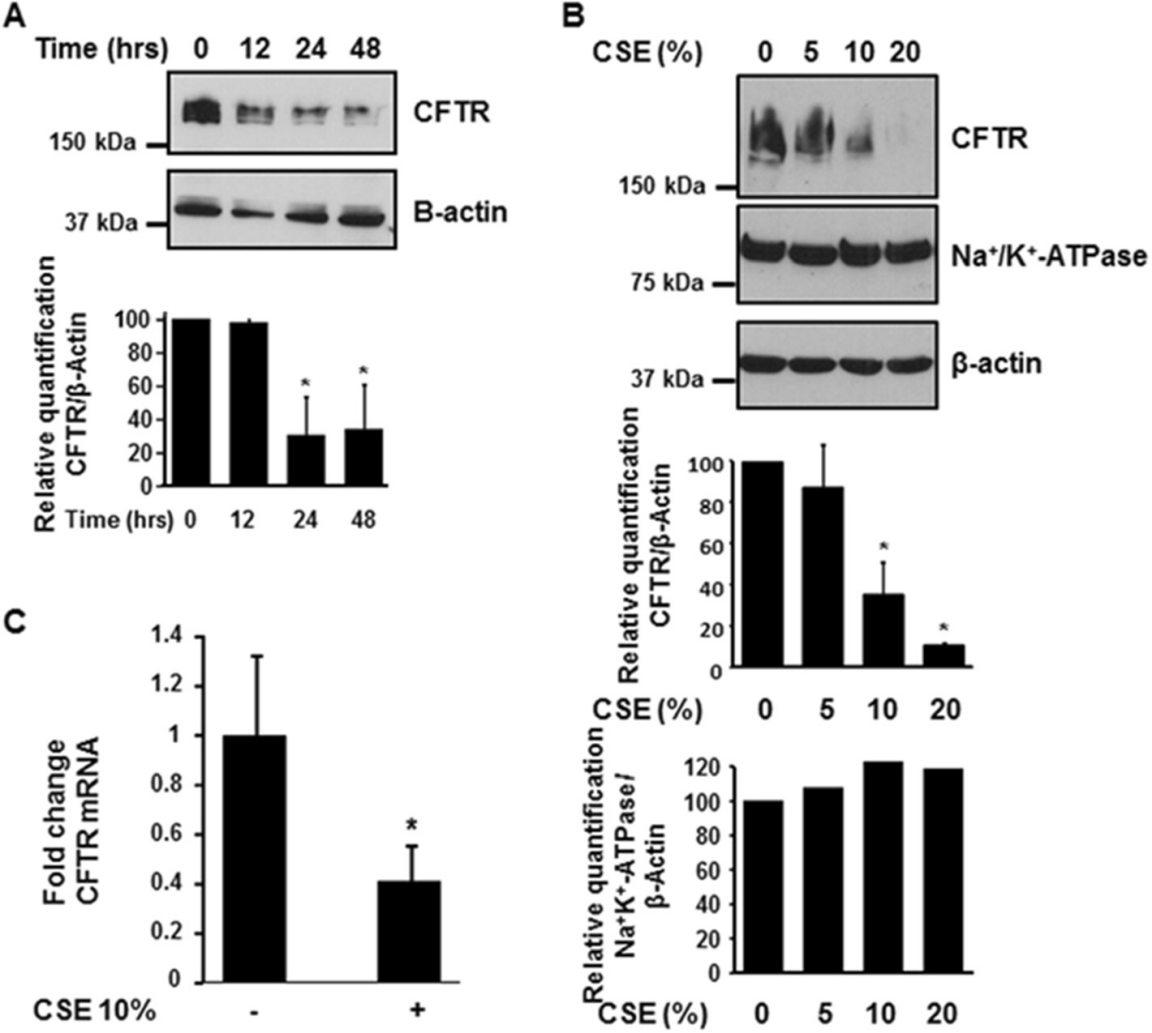
**Cigarette smoke extract (CSE) decreases the expression of CFTR but not Na**^**+**^**/K**^**+**^**-ATPase in human bronchial epithelial cells.** 16HBE14o- cells were treated with 10% CSE for up to 48 hours **(A)** or increasing concentrations of CSE prepared from commercial grade cigarettes (Camel) for 48 hours **(B)**. CFTR and Na^+^/K^+^-ATPase were detected by immunoblotting. The same amount of protein was loaded in each lane as indicated by detection of β-actin. The blots are representative of at least three independent experiments. **(C)** Detection of CFTR mRNA transcript levels using quantitative RT-PCR analysis after treatment of 16HBE14o- cells with 10% CSE for 24 hours. Results are expressed as fold change and are representative of three independent experiments. *p < 0.05.

The human airway epithelial cell line 16HBE14o- was used as a model for bronchial epithelial cells that express the CFTR protein [9]. Confluent 16HBE14o^−^ cells treated mucosally with 10% cigarette smoke extract (CSE) from commercial grade cigarettes (Camel) for 0 to 48 hours showed a time-dependent decrease in CFTR protein expression (Figure 2A). We focused on non-filtered cigarettes since CSE prepared from filtered cigarettes have limited down-regulation effect on CFTR protein when cells are exposed acutely (Additional file 1: Figure S1). We then exposed 16HBE14o- cells to increasing concentrations of CSE (5-20%) and observed a dose-dependent decrease in CFTR protein expression in response to CSE (Figure 2B). Conversely, CSE did not decrease the expression of the membrane protein Na^+^/K^+^-ATPase as seen in Figure 2B (middle panel). To assess whether CSE also affected CFTR mRNA, 16HBE14o- cells were treated with CSE. CSE down-regulated CFTR mRNA transcript levels by about 60% (Figure 2C).

It has to be noted that 16HBE14o- cells exposed to CSE exhibited no signs of toxicity as determined by the LDH cytotoxicity assay (7.5% ± 4.9 vs 6.0% ± 4.2 for control and 10% CSE, respectively).

### CFTR is decreased in the lung of GOLD 4 COPD patients

**Figure 3:**
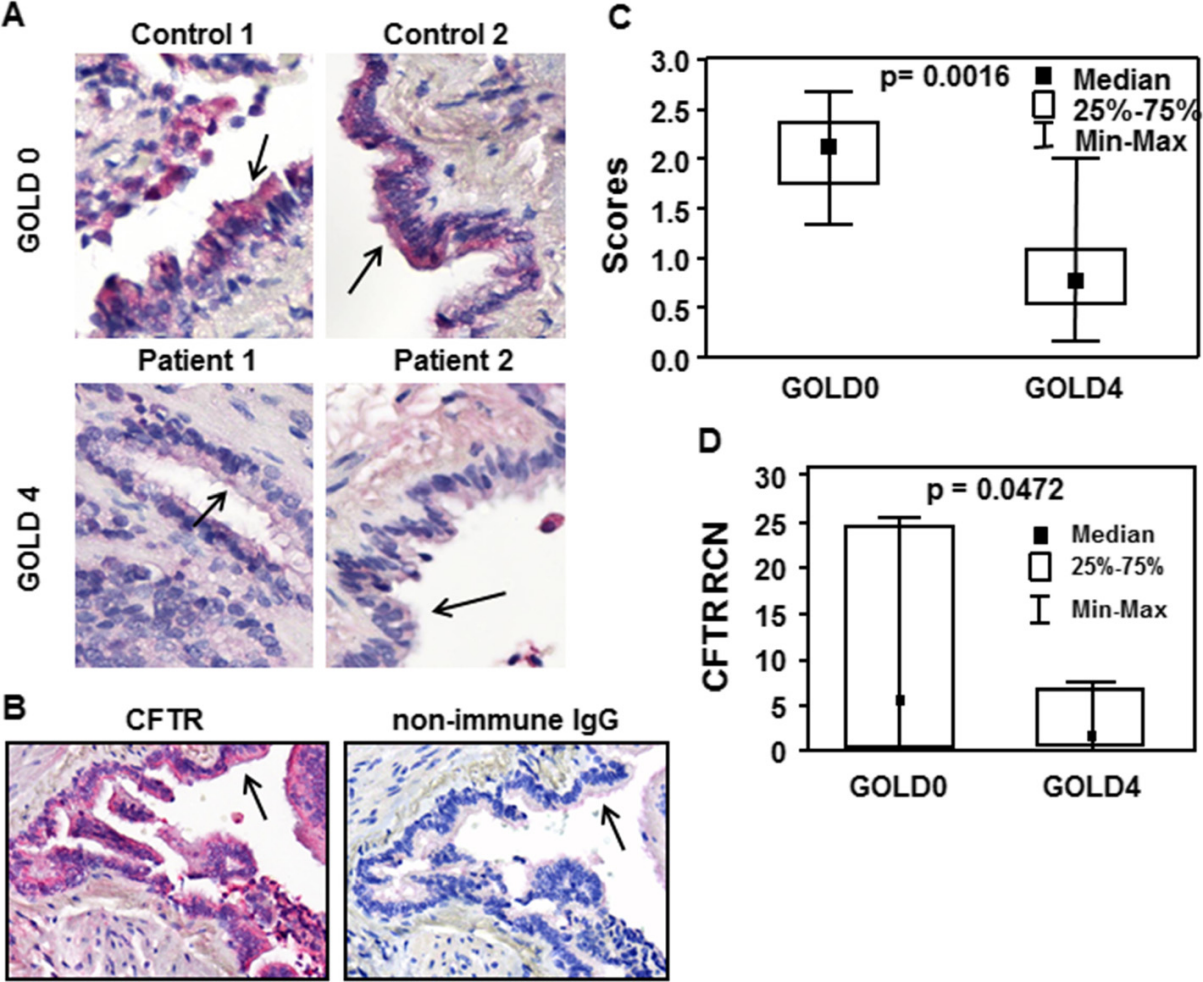
**CFTR is decreased in the lung of GOLD 4 COPD patients. (A)** CFTR protein was detected in the lung of GOLD 0 (Control 1 and 2) and GOLD 4 (Patient 1 and 2) patients. Formalin fixed paraffin embedded lung tissue sections from GOLD 0 and GOLD 4 patients were immunostained using a specific CFTR antibody (red) **(A)** or non-immune control **(B)**. **(C)** Intensity of CFTR signal was scored as described in the Methods section. **(D)** The CFTR mRNA level was measured by quantitative RT-PCR and expressed as Relative Copy Number (RCN). N = 7 for number of patients GOLD 0 and N = 8 for number of patients COPD GOLD 4. Statistically significant differences were assessed using Mann–Whitney *U* test.

We investigated the effect of long-term cigarette smoking on the expression of CFTR *in vivo*. Although all the patients included in the study had a history of cigarette smoking (except one never smoker patient in control group), they all had quit smoking when the samples were collected (except one patient in GOLD 4 group who was a current smoker). As shown in Figure 3, expression of CFTR protein was much weaker in the bronchial epithelium of the COPD GOLD 4 group when compared to the GOLD 0 group (Figure 3A). The intensity of the CFTR signal was found to be significantly reduced in bronchial epithelial cells from patients with GOLD 4 COPD (Figure 3C). No CFTR signal could be detected when non-immnune IgG was used instead of CFTR antibody (Figure 3B). Accordingly, CFTR mRNA transcript levels were significantly lower in lung samples from GOLD 4 COPD patients when compared to GOLD 0 (Figure 3D).

### Comprehensive assessment of metal content in the lung

**Figure 4:**
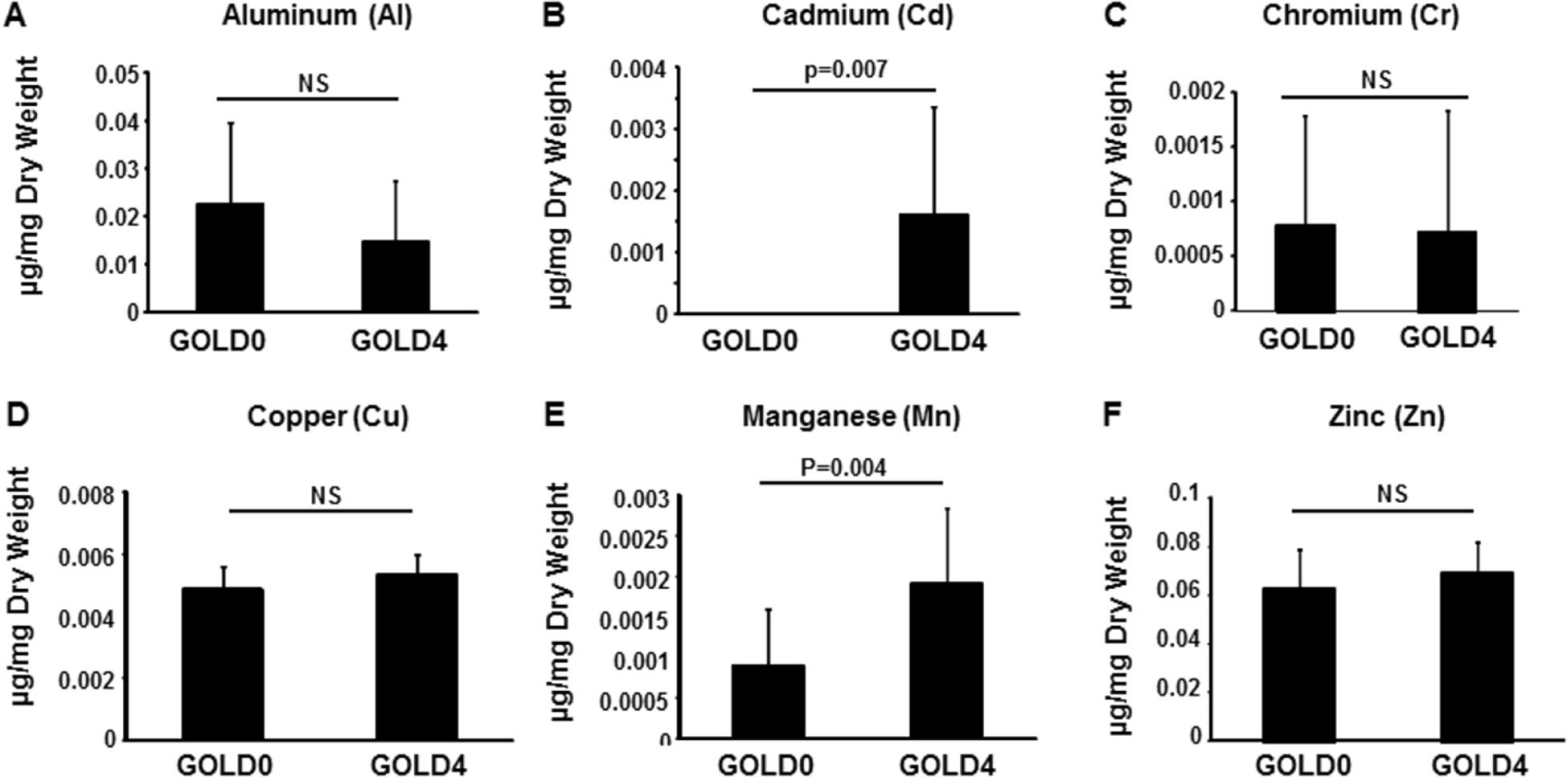
**Metal analysis of lung samples from GOLD 0 and GOLD 4 COPD patients.** The amount of aluminum **(A)**, cadmium **(B)**, chromium **(C)**, copper **(D)**, manganese **(E)**, and zinc **(F)** were measured in lung biopsies from GOLD 0 and GOLD 4 patients. Data are expressed in μg/mg dry weight tissue. N = 8 for number of patients GOLD 0 (the never smoker patient was excluded) and N = 11 for number of patients COPD GOLD 4.

We and others have reported that the pollutant metals such as arsenic and cadmium can affect the expression and function of CFTR [9,20,21]. We therefore performed a comprehensive assessment of metals present in the lung of COPD patients using ICP-AES by focusing on metals originating from cigarette smoke [22]. This analysis revealed significantly greater accumulation of cadmium and manganese in the lung of COPD GOLD 4 patients when compared to GOLD 0 patients (Figure 4B and E). It has to be noted that the amounts of cadmium present in GOLD 0 patients were below the detection level. On the other hand, no difference was seen between the amount of aluminum, chromium, copper, and zinc detected in GOLD 0 and GOLD 4 lung samples (Figure 4A, C, D, and F). Lead, nickel, selenium, and vanadium were below the detection level in all lung tissues from both patient groups.

### Role of metals present in cigarette smoke in regulation of CFTR protein

**Figure 5:**
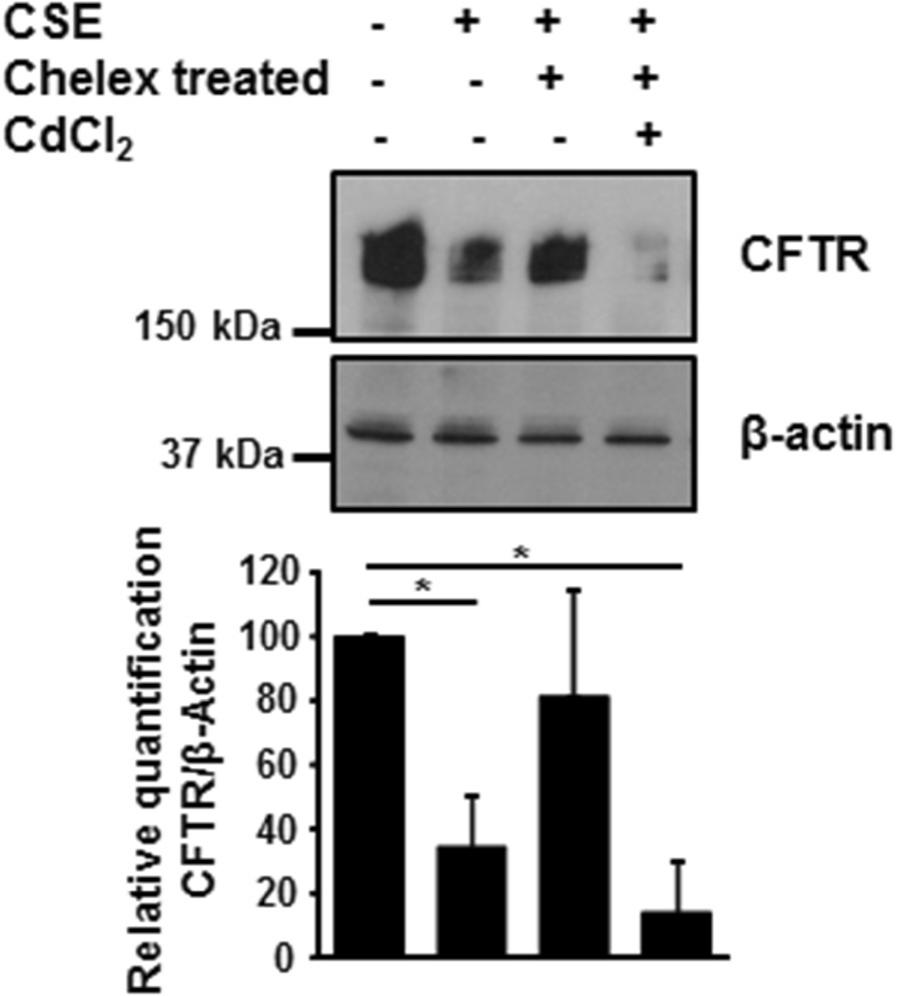
**Metals present in CSE regulate CFTR expression.** 16HBE14o- cells were incubated with 10% CSE before and after incubation with Chelex-100 beads, in absence or presence of 10 μM cadmium chloride. CFTR protein was detected by immunoblotting 48 hours after treatment. Blots are representative of at least three independent experiments. *p < 0.05.

**Figure 6:**
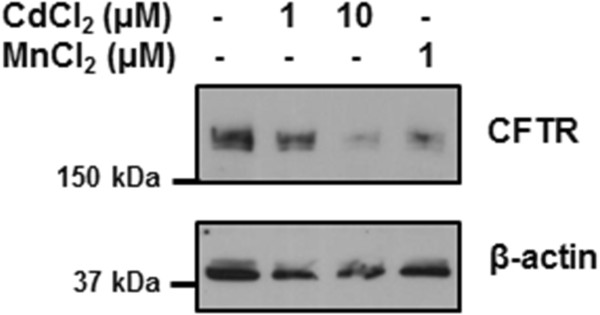
**Manganese and cadmium decrease the expression of CFTR in bronchial epithelial cells.** 16HBE14o- cells were incubated with cadmium chloride (CdCl_2_) or manganese chloride (MnCl_2_) at the doses indicated for 24 hours. CFTR protein was detected by immunobloting using a monoclonal antibody as described in Materials and Methods.

We next investigated whether metals present in cigarette smoke were involved in decrease of CFTR in bronchial epithelial cells. Metals were removed from CSE using Chelex-100 beads, which is a solid-state chelator resin that binds many divalent metals. Removal of the metals prevented the CSE-induced down-regulation of CFTR protein observed with CSE not treated with Chelex-100 beads (Figure 5, lanes 2 and 3). On the other hand, addition of cadmium to CSE treated with Chelex-100 beads resulted in a decrease in CFTR protein expression (Figure 5, lane 4). Since manganese was the other metal that was present at higher levels in the lungs of patients with COPD when compared to controls, we investigated whether manganese alone had any effect on CFTR in human bronchial epithelial cells. As observed in Figure 6, both cadmium and manganese could decrease the expression of CFTR.

## Discussion

COPD is a complex disease with multifactorial etiology. Numerous mechanisms have been implicated in the pathogenesis of COPD [23-25], yet no curative treatment has emerged, and currently there is no approach available to stop the progression of the disease. One of the main phenotypes of COPD is chronic bronchitis which is characterized by mucus secretion, chronic infection and inflammation. Recent studies showed that cigarette smoke could decrease CFTR function in nasal epithelial cells in smokers [5,8]. CFTR is a chloride channel that plays a major role in regulating ASL hydration and its activation prevents mucus accumulation in the lung [19]. However, little is known about whether CFTR expression is affected in COPD patients with a history of smoking but some studies have suggested that it could play a role in chronic bronchitis [26,27]. Our study shows that cigarette smoke decreases CFTR expression and function in human bronchial epithelial cells and that the expression of the CFTR protein is also reduced in bronchial epithelium of patients with severe (GOLD 4) COPD when compared to normal control patients (GOLD 0).

Cigarette smoking has been firmly established as the major cause of COPD, but approximately one-quarter of American adults continue to smoke, despite aggressive smoking prevention and cessation efforts [28]. On the other hand, despite the association between smoking and airway obstruction only 10 to 20% of smokers develop COPD. Here we show that CFTR protein is significantly decreased in the lung of COPD patients with severe phenotype (GOLD 4) when compared to control patients (GOLD 0). We focused on bronchial epithelial cells since CFTR is primarily expressed in those cells in the lung [29]. CFTR has also been reported to be expressed in type II pneumocytes [30]. However, due to the large destruction of the alveoli, we could not determine whether or not absence of CFTR signal was due to loss of CFTR protein or type II cells (data not shown).

CFTR function can be measured *in vivo* by measuring nasal potential differences (NPD). Cantin et al. and Clunes et al., have previously reported that current smokers have reduced CFTR function when assessing NPD [5,8]. One limitation of our study is that we were not able to measure CFTR function *in vivo* in COPD patients or control subjects due to the fact that the human samples were obtained from the Lung Tissue Research Consortium (LTRC) at the NIH and we did not have access to the patients. However, we show that chronic exposure to cigarette smoke decreases the expression of CFTR at the plasma membrane of primary human airway epithelial cells that was associated with reduction in the height of the airway surface liquid layer (see Figure 1). Our results also show that cigarette smoke has a more suppressive effect on CFTR protein than messenger RNA (see Figures 1 and 2) suggesting that strategies to restore CFTR in smokers should act at the protein level.

The composition of cigarette smoke varies markedly, especially according to the geographic origin of the tobacco leaves and contains many pollutants such as metals [22,31]. The composition of inhaled cigarette smoke by smokers depends also on whether the cigarettes smoked are filtered or not. Unfortunately, we do not know whether the patients included in this study smoked filtered or non-filtered cigarettes. Our data indicate that “acute” exposure of airway epithelial cells to cigarette smoke extract prepared from filtered cigarettes has minimal down-regulation effect on CFTR expression (Additional file 1: Figure S1). However since smokers are exposed to cigarette smoke chronically it is possible that the cumulative effect of chronic exposure to filtered cigarettes decreases CFTR expression as well. The down-regulation of CFTR expression by CSE could be recapitulated after addition of the toxic metal cadmium to Chelex-treated CSE, which demonstrated no effect on CFTR alone. Cadmium concentration has been found to be around 30 μM in the lungs of smokers and 7 μM in the aortas [32-34]. These results are in agreement with our previous study showing that cadmium, a contaminant of cigarettes, causes down-regulation of the expression and function of the CFTR chloride channel [9].

Many metals have been shown to be present in cigarette smoke, including aluminum, cadmium, chromium, copper, lead, manganese, mercury, nickel, selenium, vanadium and zinc [22]. Interestingly, only cadmium and manganese were present in higher amounts in GOLD 4 COPD patient lung samples when compared to samples from control GOLD 0 patients. In addition, the content of other metals present in cigarette smoke were similar in GOLD 0 and GOLD 4 samples even though patients in the GOLD 0 group had higher “stop years” and lower “smoking pack-year” than the GOLD 4 group (see Table 1). Manganese is an essential element but high amounts can be toxic. Manganese inhalation has been linked to chronic coughing, acute bronchitis, and decreased lung function [35]. To our knowledge, elevated accumulation of manganese in the lung of smokers has not been previously reported. It was surprising to detect such a difference in manganese, since most of our patients were ex-smokers who had quit smoking for over 7 years and the biologic half-life of manganese has been reported to be around 40 days [36]. Future studies should evaluate the contribution of manganese to lung diseases but our data shows that manganese decreases the expression of CFTR in bronchial epithelial cells (see Figure 6). On the other hand, cadmium is known to have an extremely long biological half-life (10–30 years) [37]. Interestingly, cadmium inhalation has been reported to be associated with the development of COPD in humans and animals [38-40].

## Conclusions

Our study shows that CFTR expression is decreased in the lung of patients with severe COPD and is associated with accumulation of the metals cadmium and manganese in the lung. Due to the important role played by CFTR in the lung, future studies should assess the effect of pharmacological and/or natural compounds that increase/protect CFTR in order to maintain normal lung function and prevent pathologic manifestations that could lead to chronic bronchitis.

## Competing interests

The authors declare that they have no competing interests.

## Authors’ contributions

FH contributed to the execution of the experiments, qRT-PCR, immunoblotting and immunohistochemistry. XX contributed to the execution of the experiments involving 16HBE14o- cells. GN contributed to experimental design and execution of the immunohistochemistry. DK contributed to the metal analysis. JT contributed to the ASL measurements using primary human airway epithelial cells. CDT contributed to the execution of cell surface biotinylation of CFTR using primary human airway epithelial cells. RT contributed to the design and execution of ASL measurements of the experiments using primary human airway epithelial cells. PD contributed to the background research, drafting, revision and final approval of the manuscript. JBJ contributed to the statistical analysis of the data, revision and final approval of the manuscript. DK contributed to the critical review, revision and final approval of the manuscript. PNB contributed to the manuscript preparation, revision and final approval of the manuscript. ECB contributed to the overall concept, experiment design, interpretation, and scientific context of the work performed; the drafting, critical review, revision and final approval of the manuscript. All authors read and approved the final manuscript.

## Supplementary Material

Additional file 1: Figure S1 Effect of CSE prepared from filtered and non-filtered cigarettes on CFTR expression. 16HBE14o- cells were incubated with 10% CSE prepared from filtered or non-filtered cigarettes. CFTR protein was detected by immunoblotting as described in Methods section. Blots are representative of three independent experiments. Click here for file
