# Supplementary figures and images for: Accumulation of metals in GOLD4 COPD lungs is associated with decreased CFTR levels
Source: Respir Res. 2014 Jun 23;15(1):69. doi: 10.1186/1465-9921-15-69 (PMC4106203; doi:10.1186/1465-9921-15-69)

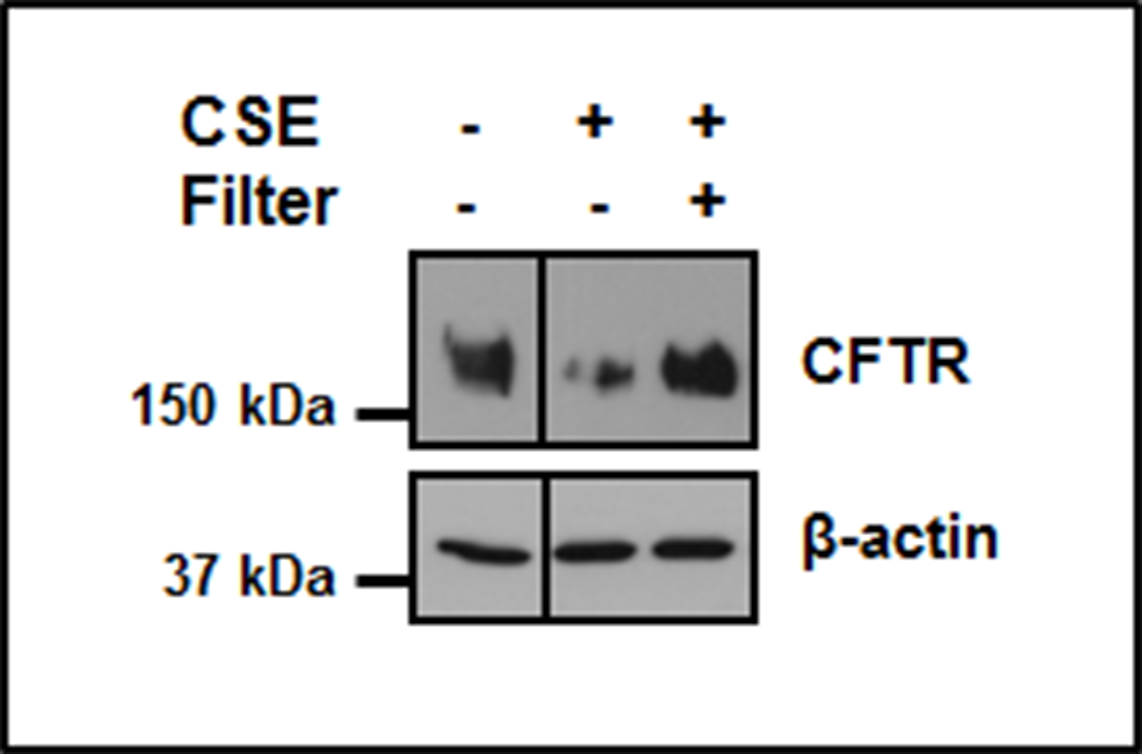

Supplement: Additional file 1: Figure S1 — Effect of CSE prepared from filtered and non-filtered cigarettes on CFTR expression. 16HBE14o- cells were incubated with 10% CSE prepared from filtered or non-filtered cigarettes. CFTR protein was detected by immunoblotting as described in Methods section. Blots are representative of three independent experiments. [file 1465-9921-15-69-S1.tiff]
